# Newly Discovered Ebola Virus Associated with Hemorrhagic Fever Outbreak in Uganda

**DOI:** 10.1371/journal.ppat.1000212

**Published:** 2008-11-21

**Authors:** Jonathan S. Towner, Tara K. Sealy, Marina L. Khristova, César G. Albariño, Sean Conlan, Serena A. Reeder, Phenix-Lan Quan, W. Ian Lipkin, Robert Downing, Jordan W. Tappero, Samuel Okware, Julius Lutwama, Barnabas Bakamutumaho, John Kayiwa, James A. Comer, Pierre E. Rollin, Thomas G. Ksiazek, Stuart T. Nichol

**Affiliations:** 1 Special Pathogens Branch, Centers for Disease Control and Prevention, Atlanta, Georgia, United States of America; 2 Scientific Resources Program, Centers for Disease Control and Prevention, Atlanta, Georgia, United States of America; 3 Center for Infection and Immunity, Mailman School of Public Health, Columbia University, New York, New York, United States of America; 4 Global AIDS Program, Centers for Disease Control and Prevention, Entebbe, Uganda; 5 Ministry of Health, Republic of Uganda, Kampala, Uganda; 6 Uganda Virus Research Institute, Entebbe, Uganda; Mount Sinai School of Medicine, United States of America

## Abstract

Over the past 30 years, *Zaire* and *Sudan ebolaviruses* have been responsible for large hemorrhagic fever (HF) outbreaks with case fatalities ranging from 53% to 90%, while a third species, *Côte d'Ivoire ebolavirus*, caused a single non-fatal HF case. In November 2007, HF cases were reported in Bundibugyo District, Western Uganda. Laboratory investigation of the initial 29 suspect-case blood specimens by classic methods (antigen capture, IgM and IgG ELISA) and a recently developed random-primed pyrosequencing approach quickly identified this to be an Ebola HF outbreak associated with a newly discovered ebolavirus species (*Bundibugyo ebolavirus*) distantly related to the *Côte d'Ivoire ebolavirus* found in western Africa. Due to the sequence divergence of this new virus relative to all previously recognized ebolaviruses, these findings have important implications for design of future diagnostic assays to monitor Ebola HF disease in humans and animals, and ongoing efforts to develop effective antivirals and vaccines.

## Author Summary

In this report we describe a newly discovered ebolavirus species which caused a large hemorrhagic fever outbreak in western Uganda. The virus is genetically distinct, differing by more than 30% at the genome level from all other known ebolavirus species. The unique nature of this virus created challenges for traditional filovirus molecular based diagnostic assays and genome sequencing approaches. Instead, we quickly determined over 70% of the virus genome using a recently developed random-primed pyrosequencing approach that allowed the rapid development of a molecular detection assay that was deployed in the disease outbreak response. This draft sequence allowed easy completion of the whole genome sequence using a traditional primer walking approach and prompt confirmation that this virus represented a new ebolavirus species. Current efforts to design effective diagnostics, antivirals and vaccines will need to take into account the distinct nature of this important new member of the filovirus family.

## Introduction

The family *Filoviridae* consists of two genera, *Marburgvirus* and *Ebolavirus*, which have likely evolved from a common ancestor [1]. The genus *Ebolavirus* is comprised of four species, *Zaire*, *Sudan*, *Reston* and *Côte d'Ivoire* (*Ivory Coast*) *ebolaviruses*, which have, with the exception of *Reston* and *Côte d'Ivoire ebolaviruses*, been associated with large hemorrhagic fever (HF) outbreaks in Africa with high case fatality (53–90%) [2]. Viruses of each species have genomes that are at least 30–40% divergent from one another, a level of diversity that presumably reflects differences in the ecologic niche they occupy and in their evolutionary history. Identification of the natural reservoir of ebolaviruses remains somewhat elusive, although recent PCR and antibody data suggest that three species of arboreal fruit bats may be carriers of *Zaire ebolavirus* [3]. No data has yet been published to suggest reservoirs for the *Sudan*, *Reston* and *Côte d'Ivoire ebolavirus* species. However, a cave-dwelling fruit bat has been recently implicated as a natural host for marburgvirus [4],[5], supporting the hypothesis that different bat species may be the reservoir hosts for the various filoviruses.

Filovirus outbreaks are sporadic, sometimes interspersed by years or even decades of no apparent disease activity. The last new species of ebolavirus was discovered 14 years ago (1994), in Cote d'Ivoire (Ivory Coast), and involved a single non-fatal case, a veterinarian who performed an autopsy on an infected chimpanzee found in the Tai Forest [6]. No further disease reports have been associated with *Côte d'Ivoire ebolavirus*, in contrast to *Zaire* and *Sudan ebolaviruses* which have each caused multiple large outbreaks over the same time period. Here, we report the isolation and characterization of a new ebolavirus that was responsible for a large hemorrhagic fever outbreak in western Uganda. This new virus, proposed name *Bundibugyo ebolavirus*, is most closely related, albeit distantly, to *Côte d'Ivoire ebolavirus*, which was somewhat unexpected, given the large geographic distance between the two countries of origin.

## Results/Discussion

**Figure 1:**
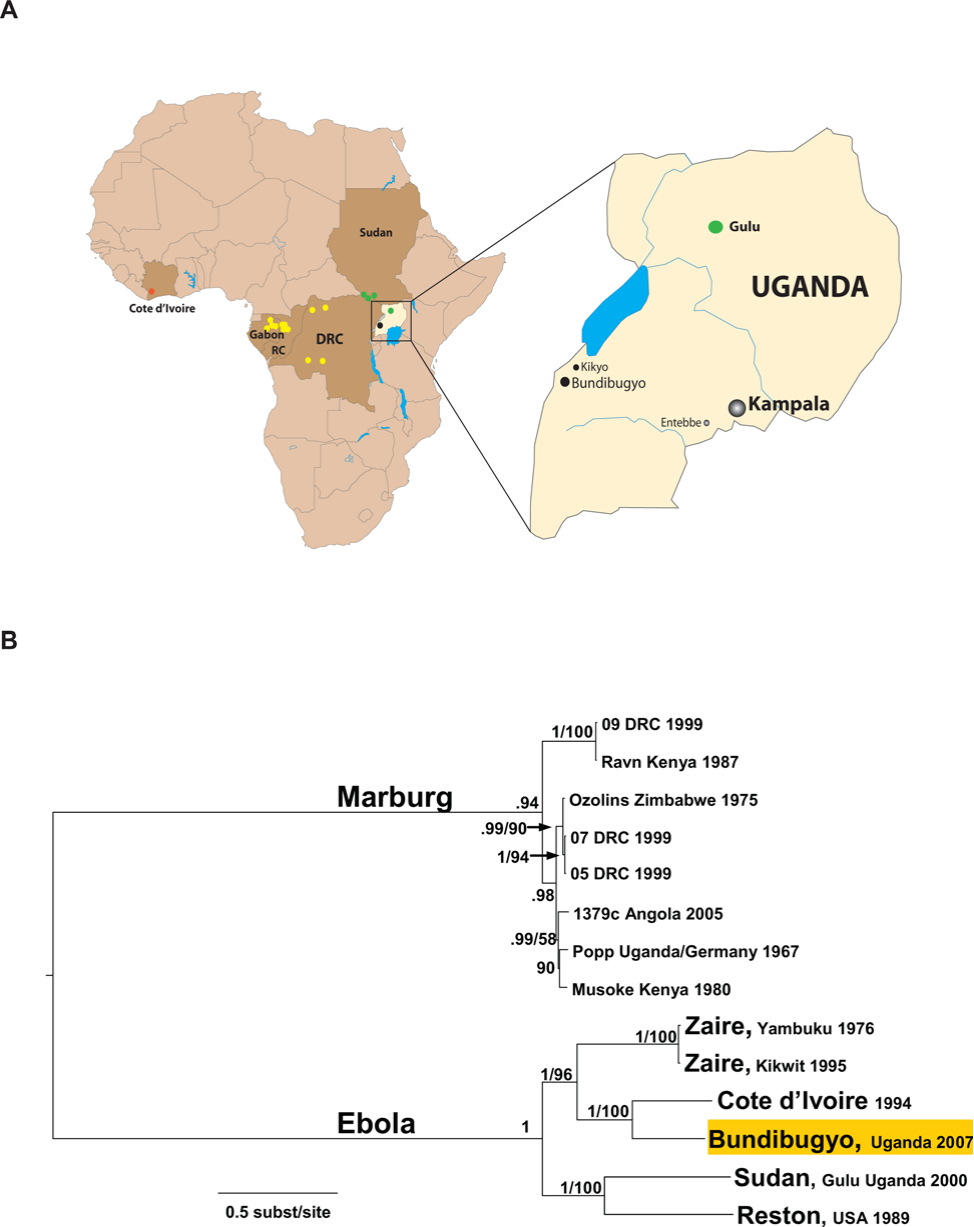
Geographic locations of Ebola HF outbreaks and phylogenetic relationships of representative filoviruses. (A) Map of Africa showing the sites of all known ebolavirus outbreaks denoted by colored circles for *Zaire ebolavirus* (yellow), *Sudan ebolavirus* (green), and *Côte d'Ivoire ebolavirus* (red). The expanded map of Uganda shows the location of the communities of Bundibugyo and Kikyo (black circles) in western Uganda, the site of the recent outbreak of *Bundibugyo ebolavirus*. Also shown on the Uganda map are the cities of Kampala (capital), Entebbe (international airport) and Gulu (the site of an outbreak of *Sudan ebolavirus* in 2000, the largest known Ebola HF outbreak on record). (B) Phylogenetic tree comparing full-length genomes of ebolavirus and marburgvirus by Bayesian analysis. Posterior probabilities greater than 0.5 and maximum likelihood bootstrap values greater than 50 are indicated at the nodes.

In late November 2007 HF cases were reported in the townships of Bundibugyo and Kikyo in Bundibugyo District, Western Uganda (Figure 1A). A total of 29 blood samples were initially collected from suspect cases and sent in two air-transport shipments to the Centers for Disease Control and Prevention (CDC) for immediate testing. Evidence of acute ebolavirus infection was detected in eight specimens using a broadly reactive ebolavirus antigen capture assay known to cross-react with the different ebolavirus species [7] and an IgM capture assay based on *Zaire ebolavirus* reagents (Table 1). The Ugandan Ministry of Health was notified on November 28, 2007. These specimens were negative when initially tested with highly sensitive real-time RT-PCR assays specific for all known *Zaire* and *Sudan ebolaviruses* and marburgviruses. However, further evidence of acute ebolavirus infection was obtained using a traditionally less sensitive (relative to the real-time RT-PCR assays) but more broadly reactive filovirus L gene-specific RT-PCR assay (1 specimen) (Table 1). Sequence analysis of the PCR fragment (400 bp of the virus L gene) revealed the reason for the initial failure of the real-time RT-PCR assays was that the sequence was distinct from the four known species of ebolavirus, although it was distantly related to *Cote d'Ivoire ebolavirus*. In total, 9 of 29 specimens showed evidence of ebolavirus infection, and all tests were negative for marburgvirus (data not shown).

**Table 1:** Ebolavirus diagnostic results of initial 29 specimens obtained from Bundibugyo District with numerical specimen numbers assigned.

Sample No.	RT-PCR	Ag	IgM	IgG	Virus Isolation	Q-RT-PCR	Ct
200706288	neg	neg	neg	neg	neg	neg	40
200706289	neg	neg	neg	neg	neg	neg	40
200706290	neg	neg	neg	neg	neg	neg	40
200706291*	**Pos**	**Pos**	neg	neg	**Pos**	**Pos**	**23.64**
200706292	neg	neg	neg	neg	neg	neg	40
200706293	neg	neg	neg	neg	neg	neg	40
200706294	neg	neg	neg	neg	neg	neg	40
200706295	neg	neg	neg	neg	neg	neg	40
200706296	neg	neg	**Pos**	**Pos**	neg	neg	40
200706297	neg	neg	**Pos**	**Pos**	neg	neg	40
200706298	neg	**Pos**	**Pos**	**Pos**	neg	**Pos**	**34.83**
200706299	neg	neg	**Pos**	**Pos**	neg	neg	40
200706300	neg	neg	neg	neg	neg	neg	40
200706301	neg	neg	neg	neg	neg	neg	40
200706302	neg	**Pos**	**Pos**	neg	neg	**Pos**	**35.01**
200706303	neg	neg	neg	neg	neg	neg	40
200706304	neg	neg	neg	neg	**Pos**	**Pos**	**38.18**
200706305	neg	neg	neg	neg	neg	neg	40
200706306	neg	neg	neg	neg	neg	neg	40
200706307	neg	neg	neg	neg	neg	neg	40
200706320	ND	**Pos**	neg	neg	**Pos**	**Pos**	**30.24**
200706321	ND	neg	neg	neg	neg	neg	40
200706322	ND	neg	neg	neg	neg	neg	40
200706323	ND	neg	neg	neg	neg	neg	40
200706324	ND	neg	neg	neg	neg	neg	40
200706325	ND	neg	neg	neg	neg	neg	40
200706326	ND	neg	neg	neg	neg	neg	40
200706327	ND	**Pos**	neg	neg	**Pos**	**Pos**	**34.41**
200706328	ND	neg	neg	neg	neg	neg	40

RT-PCR refers to results obtained from conventional PCR using the broadly reactive Filo A/B primers [16]. Ag, IgM, and IgG refer to results from ELISA-based assays [7],[13] with *Zaire ebolavirus* reagents while virus isolation refers to culture attempts on Vero E6 cells [14]. Q-RT-PCR refers to results obtained using the optimized *Bundibugyo ebolavirus* specific real-time RT-PCR assay with cycle threshold (Ct) values of positive (Pos) samples indicated in the far right column.

*: Specimen # 200706291 is the clinical sample from which prototype isolate #811250 was obtained.

Approximately 70% of the virus genome was rapidly (in less than 10 days) sequenced from total RNA extracted from a patient serum (#200706291) using a recently established metagenomics pyro-sequencing method developed at 454 Life Sciences which involves successive rounds of random DNA amplification [8]. Using the newly derived draft sequence, a real-time RT-PCR assay specific for the NP gene of this virus was quickly developed and evaluated. The assay was shown to have excellent sensitivity (Table 1), finding positive all the initial six samples that tested positive by either virus antigen capture (five specimens) or virus isolation assays (four specimens). The antigen-capture, IgM, IgG and newly designed real-time PCR assays were quickly transferred to the Uganda Virus Research Institute during the course of the outbreak to facilitate rapid identification and isolation of Ebola cases in the affected area for efficient control of the outbreak. The outbreak continued through late December, 2007, and resulted in 149 suspected cases and 37 deaths [9].

The entire genome sequence of this virus was completed using a classic primer walking sequencing approach on RNA from the reference virus isolate (#811250). The complete genome of the *Côte d'Ivoire ebolavirus* was not available, so it too was derived by a similar combination of random primed pyrosequencing and primer walking approaches. Acquisition of these sequences allowed for the first time the phylogenetic analysis of the complete genomes of representatives of all known species of Ebola and Marburg viruses. The analysis revealed that the newly discovered virus differed from the four existing ebolavirus species (Figure 1B), with approximately 32% nucleotide difference from even the closest relative, *Côte d'Ivoire ebolavirus* (Table 2). Similar complete genome divergence (35–45%) is seen between the previously characterized ebolavirus species. *Bundibugyo ebolavirus* is the proposed name for this newly identified species. This high level of genetic diversity translates to considerable amino acid differences in the encoded virus proteins. The virus surface glycoprotein, which is an important determinant of virus tropism and pathogenicity, differs between *Bundibugyo* and *Côte d'Ivoire* and *Zaire ebolaviruses* by over 27 and 35%, respectively at the amino acid level. Similarly, important virulence factors such as the immunosuppressive VP35 protein differ between *Bundibugyo* and *Côte d'Ivoire* and *Zaire ebolaviruses* by 23 and 25%, respectively. This extent of divergence will likely be reflected in significant antigenic and pathogenicity differences among these viruses.

**Table 2:** Identity (percent) matrix based on comparisons of full-length genome sequences.

	Zaire '95	Sudan '00	CdI '94	Bundi '07	Reston '89
Zaire '76	98.8	57.7	63.0	63.2	58.1
Zaire '95		57.7	63.1	63.3	58.1
Sudan '00			57.7	57.7	60.9
CdI '94				68.3	57.5
Bundi '07					57.6

The genomic sequences in the analysis are *Zaire ebolaviruses* 1976 (Genbank accession number NC_002549) and 1995 (Genbank accession number AY354458), *Sudan ebolavirus* 2000 (Genbank accession number NC_006432), *Cote d'Ivoire (CdI) ebolavirus* 1994 (Genbank accession number FJ217162), *Reston ebolavirus* 1989 (Genbank accession number NC_004161), and *Bundibugyo (Bundi) ebolavirus* 2007 (Genbank accession number FJ217161).

Current human prototype ebolavirus vaccines include *Zaire* and *Sudan ebolaviruses* [10]–[12]. Cross-protection studies will need to be done to assess whether vaccine designs will need to incorporate the *Bundibugyo ebolavirus*. The unique nature of this virus has other implications too, including screening of potential antivirals and pathogenicity studies. Retrospective analysis of case description, epidemiologic and laboratory data from the Bundibugyo outbreak are still ongoing, but it is clear that the case fatality (∼36%) associated with *Bundibugyo ebolavirus* infection is lower than that observed for *Zaire ebolavirus* (approx. 80–90%) and *Sudan ebolavirus* (approximately 50–55%) [2]. Studies in non-human primates need to be performed to compare the pathogenicity of these viruses. This investigation also highlights the power of molecular detection and characterization tools to quickly identify new pathogens, while providing a cautionary note regarding sole dependence on molecular techniques such as real-time PCR assays for detection of novel agents in biodefense or emerging disease surveillance programs.

## Materials and Methods

### *Ebolavirus* detection and virus isolation

Several diagnostic techniques were used for each sample: (i) antigen capture, IgG, and IgM assays were performed as previously described [7],[13] (ii) virus isolation attempts were performed on Vero E6 cells [14] and monitored for 14 days; (iii) RNA was extracted and tested for *Zaire* [15] and *Sudan ebolavirus* and marburgvirus [4] using real-time quantitative RT-PCR assays designed to detect all known strains of each respective virus species [the primers/probe for the *Sudan ebolavirus* assay were EboSudBMG 1(+) 5′-GCC ATG GIT TCA GGT TTG AG-3′, EboSudBMG 1(−) 5′-GGT IAC ATT GGG CAA CAA TTC A and EboSudBMG Probe 5′FAM-AC GGT GCA CAT TCT CCT TTT CTC GGA-BHQ1]; (iv) the conventional RT-PCR was performed with the filo A/B primer set as previously described [16] using Superscript III (Invitrogen) according to the manufacturer's instructions. The specimen 200706291 was selected as the reference sample for further sequence analysis.

### Genome sequencing

Pyro-sequencing was carried out utilizing the approach developed by 454 Life Sciences, and the method described by Cox-Foster et al., [8]. Subsequent virus whole genome primer walking was performed as previously described [17] but using the primers specific for *Bundibugyo ebolavirus* RT-PCR amplification. In total, the entire virus genome was amplified in six overlapping RT-PCR fragments (all primers listed 5′ to 3′): fragment A (predicted size 2.7 kb) was amplified using forward-GTGAGACAAAGAATCATTCCTG with reverse-CATCAATTGCTCAGAGATCCACC; fragment B (predicted size 3.0 kb) was amplified using forward-CCAACAACACTGCATGTAAGT with reverse-AGGTCGCGTTAATCTTCATC; fragment C (predicted size 3.5 kb) was amplified using forward-GATGGTTGAGTTACTTTCCGG with reverse-GTCTTGAGTCATCAATGCCC; fragment D (predicted size 3.1 kb) was amplified using forward-CCACCAGCACCAAAGGAC with reverse-CTATCGGCAATGTAACTATTGG; fragment E (predicted size 3.4 kb) was amplified using forward-GCCGTTGTAGAGGACACAC with reverse-CACATTAAATTGTTCTAACATGCAAG and fragment F (predicted size 3.5 kb) was amplified using forward-CCTAGGTTATTTAGAAGGGACTA with reverse-GGT AGA TGT ATT GAC AGC AAT ATC.

The exact 5′ and 3′ ends of *Bundibugyo ebolavirus* were determined by 3′ RACE from virus RNA extracted from virus infected Vero E6 cell monolayers using TriPure isolation reagent. RNAs were then polyadenylated in vitro using A-Plus poly(A) polymerase tailing kit (Epicenter Biotechnologies) following the manufacturer's instructions and then purified using an RNeasy kit (Qiagen) following standard protocols. Ten microliters of in vitro polyadenylated RNA were added as template in RT-PCR reactions, using SuperScript III One-Step RT-PCR system with Platinum *Taq* High Fidelity (Invitrogen) following the manufacturer's protocol. Two parallel RT-PCR reactions using the oligo(dT)-containing 3′RACE-AP primer (Invitrogen) mixed with 1 of 2 viral specific primers, Ebo-U 692(−) ACAAAAAGCTATCTGCACTAT and Ebo-U18269(+) CTCAGAAGCAAAATTAATGG, generated ∼700 nt long fragments containing the 3′ ends of either genomic and antigenomic RNAs. The resulting RT-PCR products were analyzed by agarose electrophoresis, and DNA bands of the correct sizes were purified using QIAquick Gel Extraction Kit (Qiagen) and sequenced using standard protocols (ABI).

The nucleotide sequence of the *Côte d'Ivoire ebolavirus* isolate RNA was initially determined using the exact same pyro-sequencing strategy as that used for *Bundibugyo ebolavirus* described above. This method generated sequence for approximately 70% of the entire genome. This draft sequence was then used to design a whole genome primer walking strategy for filling any gaps and confirming the initial sequence. The following *Côte d'Ivoire ebolavirus*-specific primers were used to generate RT-PCR fragments, designated A–F, as follows: Fragment A (predicted size 3.0 kb) was amplified using forward-GTGTGCGAATAACTATGAGGAAG and reverse-GTCTGTGCAATGTTGATGAAGG; Fragment B (predicted size 3.2 kb) was amplified using forward-CATGAAAACCACACTCAACAAC and reverse-GTTGCCTTAATCTTCATCAAGTTC; Fragment C (predicted size 3.0 kb) was amplified using forward-GGCTATAATGAATTTCCTCCAG and reverse-CAAGTGTATTTGTGGTCCTAGC; fragment D (predicted size 3.5 kb) was amplified using forward-GCTGGAATAGGAATCACAGG and reverse-CGGTAGTCTACAGTTCTTTAG; fragment E (predicted size 4.0 kb) was amplified using forward-GACAAAGAGATTAGATTAGCTATAG and reverse-GTAATGAGAAGGTGTCATTTGG; fragment F (predicted size 2.9 kb) was amplified using forward-CACGACTTAGTTGGACAATTGG and reverse-CAGACACTAATTAGATCTGGAAG; fragment G (predicted size 1.3 kb) was amplified using forward-CGGACACACAAAAAGAAWRAA and reverse-CGTTCTTGACCTTAGCAGTTC; and fragment H (predicted size 2.5 kb) was amplified using forward-GCACTATAAGCTCGATGAAGTC and reverse-TGGACACACAAAAARGARAA. A gap in the sequence contig was located between fragments C and D and this was resolved using the following primers to generate a predicted fragment of 1.5 kb: forward-CTGAGAGGATCCAGAAGAAAG and reverse-GTGTAAGCGTTGATATACCTCC. The terminal ∼20 nucleotides of the sequence were not experimentally determined but were inferred by comparing with the other known Ebola genome sequences.

### *Bundibugyo ebolavirus* real-time RT-PCR assay

The primers and probe used in the *Bundibugyo ebolavirus* specific Q-RT-PCR assay were as follows: EboU965(+): 5′-GAGAAAAGGCCTGTCTGGAGAA-3′, EboU1039(−): 5′-TCGGGTATTGAATCAGACCTTGTT-3′ and EboU989 Prb: 5′Fam-TTCAACGACAAATCCAAGTGCACGCA-3′BHQ1. Q-RT-PCR reactions were set up using Superscript III One-Step Q-RT-PCR (Invitrogen) according to the manufacturer's instructions and run for 40 cycles with a 58°C annealing temperature.

### Phylogenetic analysis

Modeltest 3.7 [18] was used to examine 56 models of nucleotide substitution to determine the model most appropriate for the data. The General Time Reversible model incorporating invariant sites and a gamma distribution (GTR+I+G) was selected using the Akaike Information Criterion (AIC). Nucleotide frequencies were A = 0.3278, C = 0.2101, G = 0.1832, T = 0.2789, the proportion of invariant sites = 0.1412, and the gamma shape parameter = 1.0593. A maximum likelihood analysis was subsequently performed in PAUP*4.0b10 [19] using the GTR+I+G model parameters. Bootstrap support values were used to assess topological support and were calculated based on 1,000 pseudoreplicates [20].

In addition, a Bayesian phylogenetic analysis was conducted in MrBayes 3.2 [21] using the GTR+I+G model of nucleotide substitution. Two simultaneous analyses, each with four Markov chains, were run for 5,000,000 generations sampling every 100 generations. Prior to termination of the run, the AWTY module was used to assess Markov Chain Monte Carlo convergence to ensure that the length of the analysis was sufficient [22]. Trees generated before the stabilization of the likelihood scores were discarded (burn in = 40), and the remaining trees were used to construct a consensus tree. Nodal support was assessed by posterior probability values (≥95 = statistical support).
